# Meta-Analysis Comparing Renal Outcomes after Transcatheter versus Surgical Aortic Valve Replacement

**DOI:** 10.1155/2019/3537256

**Published:** 2019-04-24

**Authors:** Kuldeep Shah, Zakeih Chaker, Tatiana Busu, Rishita Shah, Mohammed Osman, Fahad Alqahtani, Mohamad Alkhouli

**Affiliations:** ^1^Division of Cardiology, West Virginia School of Medicine, Morgantown, WV, USA; ^2^Department of Medicine, West Virginia School of Medicine, Morgantown, WV, USA

## Abstract

**Background:**

Acute kidney injury (AKI) is a common complication of aortic valve replacement. However, comparative on the incidence of (AKI) following transcatheter (TAVR) versus surgical valve replacement (SAVR) is sparse.

**Methods:**

We performed a meta-analysis of the randomized controlled trials (RCT) and propensity-matched observational studies comparing (A) incidence of AKI and (B) incidence of dialysis-requiring AKI at 30 days after TAVR and SAVR.

**Results:**

Twenty-six studies (20 propensity-matched studies; 6 RCTs) including 19,954 patients were analyzed. The incidence of AKI was lower after TAVR than after SAVR (7.1% vs. 12.1%, OR 0.52; 95%CI, 0.39-0.68; p<0.001, I^2^=57%), but the incidence of dialysis-requiring AKI was similar (2.8% vs. 4.1%, OR 0.78; 95%CI, 0.49-1.25; p=0.31, I^2^=70%). Similar results were observed in a sensitivity analysis including RCTs only for both AKI ([5 RCTs; 5,418 patients], 2.0% vs. 5.0%, OR 0.39; 95%CI, 0.28-0.53; p<0.001, I^2^=0%), and dialysis-requiring AKI ([2 RCTs; 769 patients]; 2.9% vs. 2.6%, OR 1.1; 95%CI, 0.47-2.58; p=0.83, I^2^=0%). However, in studies including low-intermediate risk patients only, TAVR was associated with lower incidence of AKI ([10 studies; 6,510 patients], 7.6% vs. 12.4%, OR 0.55, 95%CI 0.39-0.77, p<0.001, I^2^=57%), and dialysis-requiring AKI, ([10 studies; 12,034 patients], 2.0% vs. 3.6%, OR 0.57, 95%CI 0.38-0.85, p=0.005, I^2^=23%).

**Conclusions:**

TAVR is associated with better renal outcomes at 30 days in comparison with SAVR, especially in patients at low-intermediate surgical risk. Further studies are needed to assess the impact of AKI on long-term outcomes of patients undergoing TAVR and SAVR.

## 1. Introduction

The introduction of transcatheter aortic valve replacement (TAVR) and the continuous improvement in the outcomes of surgical aortic valve replacement (SAVR) have revolutionized the treatment of patients with severe aortic stenosis in the last decade [1, 2]. However, acute kidney injury (AKI) remains a common complication of both treatment modalities. Nonetheless, data on the incidence of AKI and dialysis-requiring AKI after TAVR vs. SAVR remain limited [3]. We performed a meta-analysis of the randomized clinical trials (RCTs) and propensity-matched (PSM) observational studies to compare renal outcomes following TAVR vs. SAVR (a) overall and (b) in subgroups of high-risk and low-intermediate risk patients.

## 2. Methods

Our review protocol was conducted in accordance with PRISMA (Preferred Reporting Items for Systematic Reviews and Meta-Analyses) reporting guidelines (Supplementary Protocol). [4] The literature search was conducted in PUBMED, MEDLINE, EMBASE, EBSCO, and Cochrane (March 2, 2018) in order to identify eligible studies using the Medical Subject Headings search terms and text word search. We also did a manual search of the reference lists of relevant studies for additional publications and when multiple publications from the same study population were found, data from the most inclusive report was used. The data was reviewed independently from full-text articles by 2 of the authors (T.B. and K.S.). Disagreements were resolved through consensus and arbitration by the senior author (M.A.). The following criteria were applied for study inclusion: (1) randomised controlled trials and propensity-matched observational studies comparing TAVR and SAVR; (2) being published in peer-reviewed journals; (3) follow-up of at least 30 days; and (4) reporting AKI or acute renal failure and/or new requirement for renal replacement therapy (dialysis-requiring AKI) as a clinical endpoint based on the valve replacement approach. Exclusion criteria we applied are (1) observational studies reporting nonpropensity-matched populations and (2) nonpublished studies (abstracts). The following study characteristics were extracted: year of publication, study design, number of patients, clinical characteristics, confounding factors, comparability between groups at baseline, outcomes, and study follow-up. The main outcomes of interest between the two interventions in this study included (1) incidence of AKI at 30 days and (2) incidence of AKI requiring dialysis at 30 days.

## 3. Data Synthesis and Analysis

The data supporting this meta-analysis are from previously reported studies and datasets, which have been cited. The processed data are reported in the article and in the supplementary files. We performed our meta-analyses using Comprehensive Meta-Analysis version 3.0 (Biostat, https://www.meta-analysis.com). We used the random effects model with the Mantel-Haenszel (MH) method for each clinical endpoint and pooled estimates of odds ratio (OR) with 95% confidence interval (CI) were calculated. We used I^2^ index, tau squared, and the Q-test* P* value to examine heterogeneity among individual study effect sizes. To reduce the risk of bias, we undertook independent pooling of data from RCTs and PSM observational studies. In order to formally assess publication bias we prepared funnel plots and Egger's linear regression test of funnel plot asymmetry (eFigures 1 and 2). All pooled estimates are displayed with a 95% confidence interval (CI).* P* values were considered statistically significant at less than 0.05. We also performed sensitivity analysis to investigate potential sources of inconsistency, including removal of nonrandomized studies. Forest plots were generated to show the relative effect size of TAVR and SAVR for each clinical outcome. Potential sources of heterogeneity were investigated using meta-regression techniques; factors analyzed in the metaregression included age, sex, diabetes, prior stroke, chronic renal insufficiency, vascular disease, and atrial fibrillation (eFigure 3). We followed standard protocol for performing meta-analysis as in our previous publication. The endpoint of interest in this study is renal outcomes which were not described in our previous papers making this study unique. [5, 6]

## 4. Results

**Figure 1:**
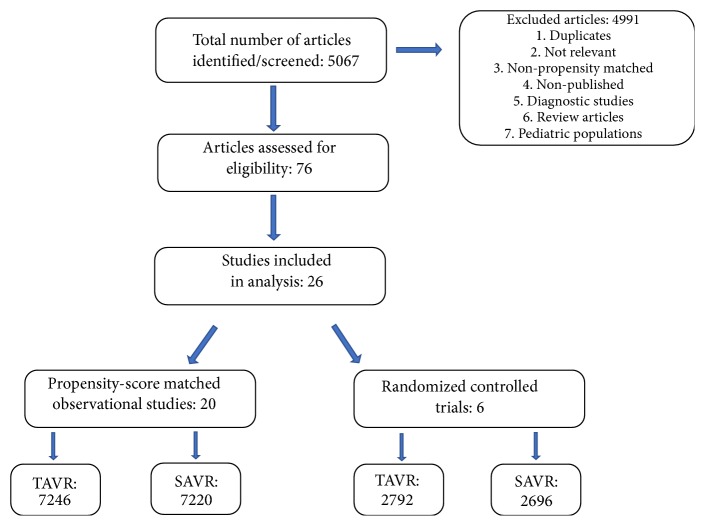
Flow chart of the meta-analysis. TAVR: transcatheter aortic valve replacement, SAVR: surgical aortic valve replacement.

A total of 5,067 potentially relevant citations were identified and screened (Figure 1). After removal of duplicated studies, we retrieved 76 full-text articles for evaluation, of which 26 satisfied the selection criteria. A total of 20 PSM observational studies and 6 RCTs were included in the meta-analysis (Figure 1). All eligible studies were in the English Language. The baseline characteristics of the patients in the included studies are summarized in Table 1. The 26 studies enrolled a total of 19,954 patients; 10,038 (50.3%) in the TAVR group and 9,916 (49.7%) in the SAVR group. Sample sizes ranged from 28 to 4732 patients. Mean age was 79.1±5.8 and 78.1±5.9 years in the TAVR and SAVR groups, respectively (p=0.53). There was no significant difference in the prevalence of key morbidities between the two groups including chronic renal insufficiency (25.9% in the TAVR group vs. 25.3% in the SAVR group, p=0.94) (Table 1). Detailed baseline characteristics of individual studies included in our meta-analysis are illustrated in eTable 1. [7–32]

### 4.1. Meta-Analysis of RCT and PSM Studies

**Figure 2:**
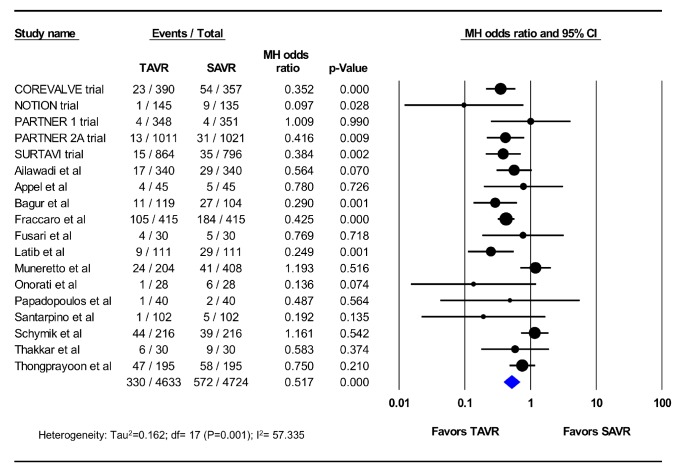
Pooled effect estimates for 30-day acute kidney injury according to the type of aortic valve replacement procedure. TAVR: transcatheter aortic valve replacement, SAVR: surgical aortic valve replacement.

**Figure 3:**
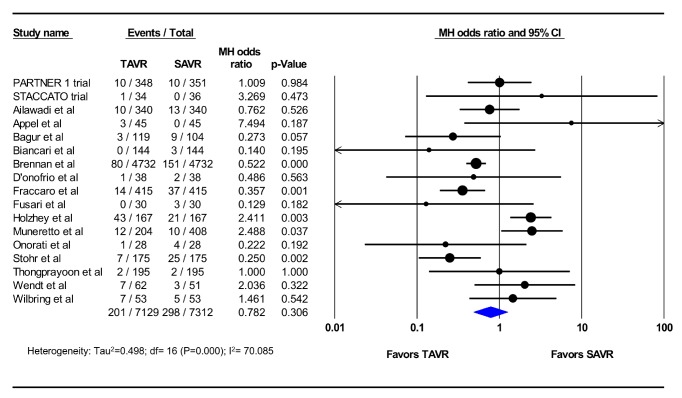
Pooled effect estimates for 30-day renal replacement therapy according to the type of aortic valve replacement procedure. TAVR: transcatheter aortic valve replacement, SAVR: surgical aortic valve replacement.

Eighteen studies (5 RCTs and 13 PSM observational studies; 4,633 TAVR patients; 4,724 SAVR patients) reported the incidence of AKI at 30 days. The pooled estimated incidence of AKI among these studies was 7.1% after TAVR and 12.1% after SAVR (OR 0.52; 95%CI, 0.39-0.68; p<0.001) (I^2^=57%) (Figure 2). Seventeen studies (2 RCTs and 15 PSM observational studies; 7,129 TAVR patients; 7,312 SAVR patients) reported the incidence of dialysis-requiring AKI at 30 days, which was similar between patients who underwent TAVR and those who underwent SAVR (2.8% vs. 4.1%, OR 0.78; 95% CI, 0.49-1.25; p=0.31) (I^2^=70%) (Figure 3). In the meta-regression, age, sex, and the diabetes, prior stroke, chronic renal insufficiency, vascular disease, and atrial fibrillation did not explain the observed heterogeneity between the studies (Supplementary Figures).

### 4.2. Meta-Analysis of RCT Only

**Figure 4:**
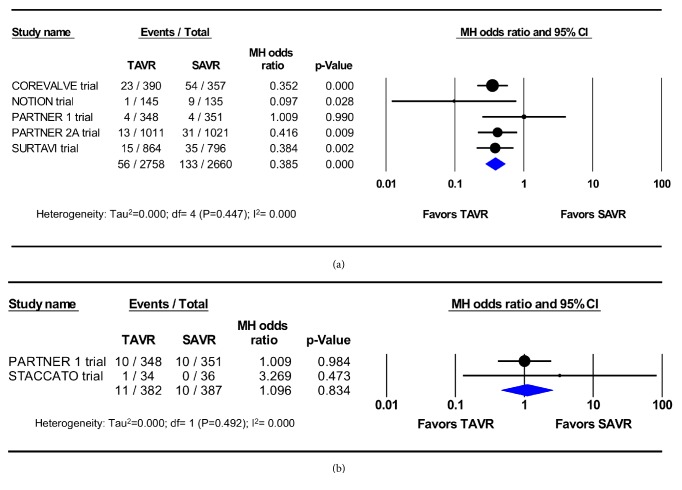
Pooled effect estimates for 30-day acute kidney injury and renal replacement therapy according to the type of aortic valve replacement procedure in the randomized controlled trials. TAVR: transcatheter aortic valve replacement, SAVR: surgical aortic valve replacement.

A sensitivity analysis was performed by excluding PSM studies and restricting the meta-analysis to RCTs only. Similar to the original analysis, this meta-analysis showed significantly lower incidence of AKI after TAVR than after SAVR (5 RCTs, 5,418 patients, 2.0% vs. 5.0%, OR 0.39; 95%CI, 0.28-0.53; p<0.001) (Figure 4(a)), but comparable rates of dialysis-requiring AKI (2 studies; 769 patients; 2.9% vs. 2.6%, OR 1.1; 95% CI, 0.47-2.58; p=0.83) (Figures 4(a) and 4(b)). No heterogeneity among these trials was observed (I^2^=0%).

### 4.3. Meta-Analysis Stratified by Surgical Risk

**Figure 5:**
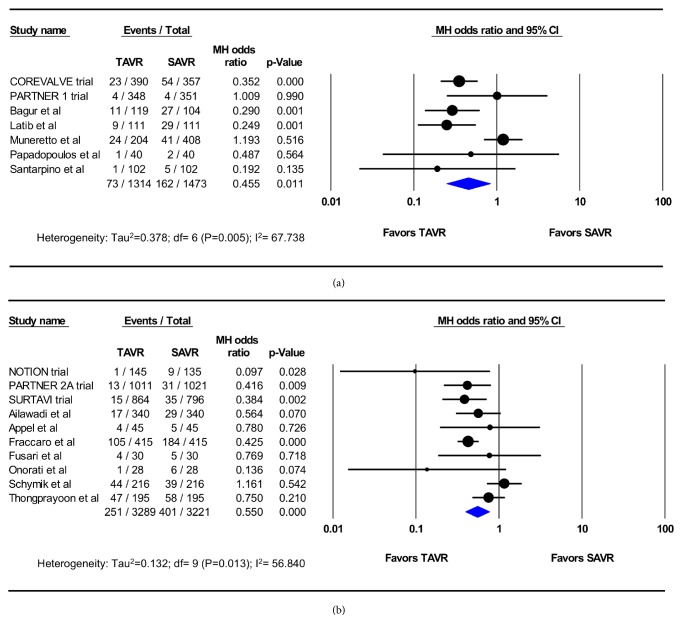
Pooled effect estimates for 30-day acute kidney injury according to the type of aortic valve replacement procedure in the randomized controlled trials. TAVR: transcatheter aortic valve replacement, SAVR: surgical aortic valve replacement.

**Figure 6:**
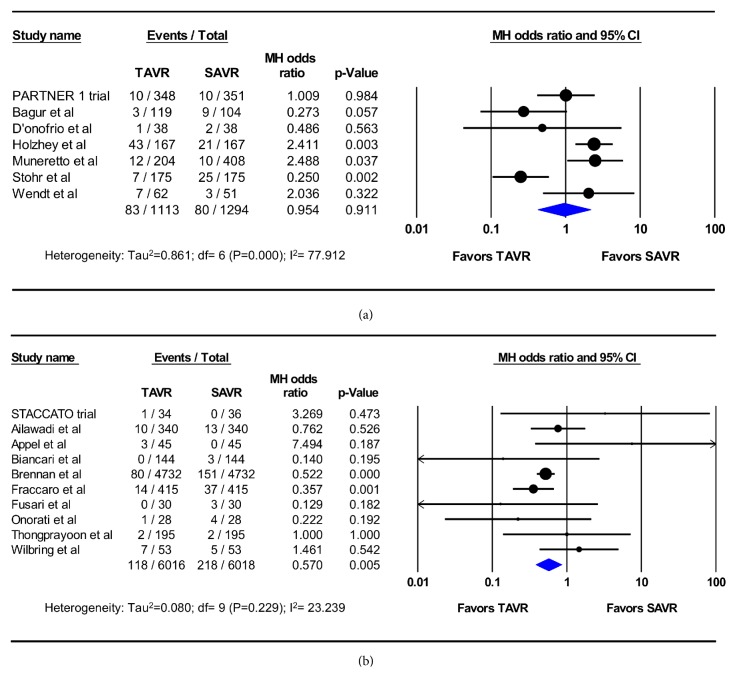
Pooled effect estimates for 30-day renal replacement therapy according to the type of aortic valve replacement procedure and surgical risk. TAVR: transcatheter aortic valve replacement, SAVR: surgical aortic valve replacement.

A secondary analysis was performed to compare the pooled incidence of AKI and dialysis-requiring AKI among patients who are at high surgical risk and those at low-intermediate surgical risk.

(A) Renal outcomes in high-surgical risk patients: seven studies including 2,787 patients reported the incidence of AKI in high-surgical risk patients who underwent TAVR vs. SAVR. In these studies, TAVR was associated with lower pooled incidence of AKI (5.5% vs. 11%, OR 0.45, 95%CI 0.25-0.83, p=0.01, I^2^=68%) (Figure 5(a)). Seven studies including 2,407 patients reported the incidence of dialysis-requiring AKI after valve replacement in high-surgical risk patients. In these studies, there was no significant difference in the pooled incidence of dialysis-requiring AKI between TAVR and SAVR (7.4% vs. 6.2%, OR 0.95, 95%CI 0.42-2.16, p=0.91, I^2^=78%) (Figure 6(a)).

(B) Renal outcomes in low-intermediate surgical risk patients: ten studies including 6,510 patients reported the incidence of AKI following TAVR vs. SAVR in low-intermediate surgical risk patients that compared with SAVR, TAVR was associated with lower pooled incidence of AKI (7.6% vs. 12.4%, OR 0.55, 95%CI 0.39-0.77, P<0.001, I^2^=57%) (Figure 5(b)). Also, in the ten studies (n=12,034 patients) that reported the incidence of dialysis-requiring AKI in this cohort of patient, TAVR was associated with significantly lower incidence of dialysis-requiring AKI compared with SAVR (2.0% vs. 3.6%, OR 0.57, 95%CI 0.38-0.85, p=0.005, I^2^=23%) (Figure 6(b)).

## 5. Discussion

The major findings of the current investigation are as follows. (1) TAVR is associated with lower rates of AKI compared with SAVR, and this was consistent in the overall analysis, in a sensitivity analysis including RCTs only, and in subanalyses of high-risk and low-intermediate risk patients. (2) The risk of dialysis-requiring AKI appears to be comparable after TAVR vs. SAVR. However, the pooled incidence of dialysis-requiring AKI was significantly lower after TAVR than after SAVR in a subgroup of low-intermediate risk patients.

Patients with severe aortic stenosis are characteristically older and have many comorbidities including a high prevalence of chronic renal insufficiency. Cardiac surgery operations including SAVR are associated with significant risk of AKI and AKI requiring dialysis [33, 34]. Transcatheter aortic valve replacement was introduced as an effective alternative to surgery in high-prohibitive risk patients but later expanded into young and lower risk patient cohorts. Nonetheless, both the preoperative work-up and the TAVR procedure itself carry a significant risk of AKI due to contrast medium usage, and the high prevalence of atherosclerotic risk factors among patients submitted for TAVR. Whether TAVR is associated with lower risk of AKI and AKI requiring dialysis than SAVR has not been well studied. In the pivotal PARTNER-1 trial, no difference in the rate of AKI was observed between TAVR and SAVR. [7] Subsequent RCTs showed lower rates of AKI after TAVR compared with SAVR. We hence performed a systematic review and a meta-analysis to synthesize the best available evidence on renal outcomes following TAVR and SAVR.

Our meta-analysis showed that TAVR is associated with about 50% reduction in the incidence of AKI compared with SAVR, but a similar rate of dialysis-requiring AKI between the two modalities overall. These findings have important prognostic implications and deserve more scrutiny for several reasons. (1) There is ample evidence that even AKI not requiring dialysis is associated with substantial negative impact on long-term outcomes [35–41]. (2) The risk of AKI and dialysis-requiring AKI may be more modifiable in patients undergoing TAVR. The advances in 3D echocardiography and the refinements in TAVR techniques have allowed the introduction of the ‘Reno-protective TAVR' concept [42–46]. This concept along with the wide adoption of moderate sedation in TAVR procedures has the potential to further reduce postprocedural renal insufficiency although this has not yet been studied in a prospective fashion [47]. In contrast, the risk of AKI after SAVR may be more related to the patient risk profile than to modifiable procedural factors as surgical techniques in SAVR have not differed significantly in the last decade. (3) In our subanalysis of patients who are at low-intermediate surgical risk, TAVR was associated with a significant reduction not only in AKI but also in AKI requiring dialysis. In light of the continuous expansion of TAVR to lower risk populations, the impact of the TAVR on improving renal outcomes in these patients warrants more investigation [48].

## 6. Limitations

Our study has several limitations: (1) there are only few RCTs comparing TAVR with SAVR. Hence, we included observational studies in our meta-analysis. However, we limited our inclusion of observational studies to those with propensity score matched comparisons. While this can introduce heterogeneity into our analysis, our sensitivity analysis including RCTs only yielded similar results to the overall meta-analysis. (2) The definition of AKI varies among the studies, but those definitions were maintained the same in the same study for comparison between TAVR and SAVR, and hence the results are comparable.

## 7. Conclusions

TAVR procedure has significantly lower rates of AKI compared to SAVR but similar rates of AKI requiring renal replacement therapy. AKI has short and long-term effects on outcomes and survival; hence every effort should be made to reduce the incidence of AKI.

## Figures and Tables

**Table 1:** 

Baseline Characteristics	TAVR (N=10,038)	SAVR (N=9,916)	p-value
Age (years)	79.1 ± 5.8	78.06 ± 5.9	0.528
Male	49.17%	50.16%	0.798
Coronary artery disease	57.7%	52.99%	0.606
Chronic kidney disease (GFR<60 mL/min)	25.9%	25.3%	0.943
Diabetes mellitus	30.0%	31.2%	0.768
Atrial fibrillation	28.6%	28.6%	0.991
Chronic obstructive pulmonary disease	23.0%	22.7%	0.927
Frailty	28.4%	27.5%	0.957
Left ventricular ejection fraction	56.41 ± 6.6	55.3 ± 9.0	0.701
Pulmonary hypertension	21.1%	18.7%	0.729
Peripheral vascular disease	24.4%	22.6%	0.630
Prior stroke or transient ischemic attack	16.5%	15.8%	0.838
NYHA III or IV	71.4%	67.73%	0.515
Prior coronary artery bypass graft	40.9%	31.8%	0.445
STS score	6.6 ± 2.9	6.1 ± 2.3	0.590
Euro SCORE	17.1 ± 8.3	15.0 ± 6.3	0.357

## Data Availability

The data used to support the findings of this study are available from the corresponding author upon request.

## Supplementary Materials

Supplementary Materials eFigure 1: Funnel plot of the meta-analysis of the published studies reporting 30-day acute kidney injury in patients undergoing TAVR versus SAVR. TAVR: transcatheter aortic valve replacement; SAVR: surgical aortic valve replacement. eFigure 2: Funnel plot of the meta-analysis of the published studies reporting 30-day renal replacement therapy in patients undergoing TAVR versus SAVR. TAVR: transcatheter aortic valve replacement; SAVR: surgical aortic valve replacement. eFigure 3: Meta regression for age, gender, previous stroke, peripheral arterial disease, diabetes, chronic kidney disease, atrial fibrillation, and acute kidney injury and renal replacement therapy in patients undergoing TAVR versus SAVR. TAVR: transcatheter aortic valve replacement; SAVR: surgical aortic valve replacement.

## References

[1] Holmes D. R., MacK M. J., Kaul S. (2012). 2012 ACCF/AATS/SCAI/STS expert consensus document on transcatheter aortic valve replacement: developed in collaboration with the american heart association, american society of echocardiography, european association for cardio-thoracic surgery, heart failure society of america, mended hearts, society of cardiovascular anesthesiologists. *The Annals of Thoracic Surgery*.

[2] Nishimura R. A., Otto C. M., Bonow R. O. (2014). AHA/ACC guideline for the management of patients with valvular heart disease: a report of the American College of Cardiology/American Heart Association Task Force on Practice Guidelines. *Journal of the American College of Cardiology*.

[3] Kumar N., Garg N. (2018). Acute kidney injury after aortic valve replacement in a nationally representative cohort in the USA. *Nephrology Dialysis Transplantation*.

[4] Moher D., Liberati A., Tetzlaff J., Altman D. G. (2010). Preferred reporting items for systematic reviews and meta-analyses: the PRISMA statement. *International Journal of Surgery*.

[5] Shah K., Chaker Z., Busu T. (2018). Meta-analysis comparing the frequency of stroke after transcatheter versus surgical aortic valve replacement. *American Journal of Cardiology*.

[6] Busu T., Alqahtani F., Badhwar V., Cook C. C., Rihal C. S., Alkhouli M. (2018). Meta-analysis comparing transcatheter and surgical treatments of paravalvular leaks. *American Journal of Cardiology*.

[7] Smith C. R., Leon M. B., Mack M. J. (2011). Transcatheter versus surgical aortic-valve replacement in high-risk patients. *The New England Journal of Medicine*.

[8] Nielsen H. H. M., Klaaborg K. E., Nissen H. (2012). A prospective, randomised trial of transapical transcatheter aortic valve implantation vs. surgical aortic valve replacement in operable elderly patients with aortic stenosis: The STACCATO trial. *EuroIntervention*.

[9] Adams D. H., Popma J. J., Reardon M. J. (2014). Transcatheter aortic-valve replacement with a self-expanding prosthesis. *The New England Journal of Medicine*.

[10] Thyregod H. G., Steinbruchel D. A., Ihlemann N. (2015). Transcatheter versus surgical aortic valve replacement in patients with severe aortic valve stenosis: 1-year results from the all-comers notion randomized clinical trial. *Journal of the American College of Cardiology*.

[11] Leon M. B., Smith C. R., Mack M. J. (2016). Transcatheter or surgical Aortic-valve replacement in intermediate-risk patients. *The New England Journal of Medicine*.

[12] Reardon M. J., Van Mieghem N. M., Popma J. J. (2017). Surgical or transcatheter aortic-valve replacement in intermediate-risk patients. *The New England Journal of Medicine*.

[13] Bagur R., Webb J. G., Nietlispach F. (2010). Acute kidney injury following transcatheter aortic valve implantation: Predictive factors, prognostic value, and comparison with surgical aortic valve replacement. *European Heart Journal*.

[14] Fusari M., Bona V., Muratori M. (2012). Transcatheter vs. surgical aortic valve replacement: A retrospective analysis assessing clinical effectiveness and safety. *Journal of Cardiovascular Medicine*.

[15] Stöhr R., Dohmen G., Herpertz R. (2011). Thirty-day outcome after transcatheter aortic valve implantation compared with surgical valve replacement in patients with high-risk aortic stenosis: A matched comparison. *Coronary Artery Disease*.

[16] Holzhey D. M., Shi W., Rastan A., Borger M. A., Hänsig M., Mohr F. W. (2012). Transapical versus conventional aortic valve replacement - A propensity-matched comparison. *Heart Surgery Forum*.

[17] Appel C.-F., Hultkvist H., Nylander E. (2012). Transcatheter versus surgical treatment for aortic stenosis: Patient selection and early outcome∗. *Scandinavian Cardiovascular Journal*.

[18] Latib A., Maisano F., Bertoldi L. (2012). Transcatheter vs surgical aortic valve replacement in intermediate- surgical-risk patients with aortic stenosis: A propensity score-matched case-control study. *American Heart Journal*.

[19] D'Onofrio A., Messina A., Lorusso R. (2012). Sutureless aortic valve replacement as an alternative treatment for patients belonging to the “gray zone” between transcatheter aortic valve implantation and conventional surgery: A propensity-matched, multicenter analysis. *The Journal of Thoracic and Cardiovascular Surgery*.

[20] Wilbring M., Tugtekin S.-M., Alexiou K., Simonis G., Matschke K., Kappert U. (2013). Transapical transcatheter aortic valve implantation vs conventional aortic valve replacement in high-risk patients with previous cardiac surgery: A propensity-score analysis. *European Journal of Cardio-Thoracic Surgery*.

[21] Papadopoulos N., Schiller N., Fichtlscherer S. (2014). Propensity matched analysis of longterm outcomes following transcatheter based aortic valve implantation versus classic aortic valve replacement in patients with previous cardiac surgery. *Journal of Cardiothoracic Surgery*.

[22] Santarpino G., Pfeiffer S., Jessl J. (2015). Clinical outcome and cost analysis of sutureless versus transcatheter aortic valve implantation with propensity score matching analysis. *American Journal of Cardiology*.

[23] Schymik G., Heimeshoff M., Bramlage P. (2015). A comparison of transcatheter aortic valve implantation and surgical aortic valve replacement in 1,141 patients with severe symptomatic aortic stenosis and less than high risk. *Catheterization and Cardiovascular Interventions*.

[24] Muneretto C., Alfieri O., Cesana B. M. (2015). A comparison of conventional surgery, transcatheter aortic valve replacement, and sutureless valves in “real-world” patients with aortic stenosis and intermediate- to high-risk profile. *The Journal of Thoracic and Cardiovascular Surgery*.

[25] Wendt D., Al-Rashid F., Kahlert P. (2015). Conventional aortic valve replacement or transcatheter aortic valve implantation in patients with previous cardiac surgery. *Journal of Cardiology*.

[26] Thakkar B., Patel A., Mohamad B. (2016). Transcatheter aortic valve replacement versus surgical aortic valve replacement in patients with cirrhosis. *Catheterization and Cardiovascular Interventions*.

[27] Thongprayoon C., Cheungpasitporn W., Srivali N. (2016). AKI after transcatheter or surgical aortic valve replacement. *Journal of the American Society of Nephrology*.

[28] Biancari F., Barbanti M., Santarpino G. (2016). Immediate outcome after sutureless versus transcatheter aortic valve replacement. *Heart and Vessels*.

[29] Onorati F., DOnofrio A., Biancari F. (2016). Results of surgical aortic valve replacement and transapical transcatheter aortic valve replacement in patients with previous coronary artery bypass grafting. *Interact Cardiovasc Thorac Surg*.

[30] Fraccaro C., Tarantini G., Rosato S. (2016). Early and Midterm Outcome of Propensity-Matched Intermediate-Risk Patients Aged ≥80 Years with Aortic Stenosis Undergoing Surgical or Transcatheter Aortic Valve Replacement (from the Italian Multicenter OBSERVANT Study). *American Journal of Cardiology*.

[31] Ailawadi G., LaPar D. J., Speir A. M. (2016). Contemporary costs associated with transcatheter aortic valve replacement. *The Annals of Thoracic Surgery*.

[32] Brennan J. M., Thomas L., Cohen DJ. (2017). Transcatheter versus surgical aortic valve replacement: propensity-matched comparison. *Journal of the American College of Cardiology*.

[33] Reents W., Hilker M., Börgermann J. (2014). Acute kidney injury after on-pump or off-pump coronary artery bypass grafting in elderly patients. *The Annals of Thoracic Surgery*.

[34] Vives M., Wijeysundera D., Marczin N., Monedero P., Rao V. (2014). Cardiac surgery-associated acute kidney injury. *Interactive CardioVascular and Thoracic Surgery*.

[35] Liao Y.-B., Deng X.-X., Meng Y. (2017). Predictors and outcome of acute kidney injury after transcatheter aortic valve implantation: A systematic review and meta-analysis. *EuroIntervention*.

[36] Chatani K., Abdel-Wahab M., Wübken-Kleinfeld N. (2015). Acute kidney injury after transcatheter aortic valve implantation: Impact of contrast agents, predictive factors, and prognostic importance in 203 patients with long-term follow-up. *Journal of Cardiology*.

[37] Konigstein M., Ben-Assa E., Banai S. (2015). Periprocedural bleeding, acute kidney injury, and long-term mortality after transcatheter aortic valve implantation. *Canadian Journal of Cardiology*.

[38] Ma M., Gao W. D., Gu Y. F., Wang Y. S., Zhu Y., He Y. (2018). Clinical effects of acute kidney injury after transcatheter aortic valve implantation: a systematic review and meta-analysis. *Internal and Emergency Medicine*.

[39] Kliuk-Ben Bassat O., Finkelstein A., Bazan S. (2018). Acute kidney injury after transcatheter aortic valve implantation and mortality risk-long-term follow-up. *Nephrology Dialysis Transplantation*.

[40] Nunes Filho A. C. B., Katz M., Campos C. M. (2018). Impact of acute kidney injury on short- and long-term outcomes after transcatheter aortic valve implantation. *Revista Española de Cardiología*.

[41] Muñoz-García A. J., Muñoz-García E., Jiménez-Navarro M. F. (2015). Clinical impact of acute kidney injury on short- and long-term outcomes after transcatheter aortic valve implantation with the CoreValve prosthesis. *Journal of Cardiology*.

[42] Higuchi R., Tobaru T., Hagiya K. (2018). Renoprotective transcatheter aortic valve implantation without contrast media. *International Heart Journal*.

[43] van Mourik M. S., van Kesteren F., Planken R. N. (2018). Short versus conventional hydration for prevention of kidney injury during pre-TAVI computed tomography angiography. *Netherlands Heart Journal*.

[44] Elkaryoni A., Nanda N. C., Baweja P. (2018). Three-dimensional transesophageal echocardiography is an attractive alternative to cardiac multi-detector computed tomography for aortic annular sizing: Systematic review and meta-analysis. *Journal of Echocardiography*.

[45] Renker M., Varga-Szemes A., Schoepf U. J. (2016). A non-contrast self-navigated 3-dimensional MR technique for aortic root and vascular access route assessment in the context of transcatheter aortic valve replacement: proof of concept. *European Radiology*.

[46] Pershad A., Fraij G., Girotra S. V., Fang H. K., Gellert G. (2015). TEE-guided transcatheter aortic valve implantation with "Zero Contrast" - A viable alternative for patients with chronic kidney disease. *The Journal of Invasive Cardiology*.

[47] Hyman M. C., Vemulapalli S., Szeto W. Y. (2017). Conscious Sedation Versus General Anesthesia for Transcatheter Aortic Valve Replacement:Insights from the National Cardiovascular Data Registry Society of Thoracic Surgeons/American College of Cardiology Transcatheter Valve Therapy Registry. *Circulation*.

[48] Holmes D. R., Nishimura R. A., Grover F. L. (2016). Annual Outcomes With Transcatheter Valve Therapy: From the STS/ACC TVT Registry. *The Annals of Thoracic Surgery*.

