# Supplementary material for: Meta-Analysis Comparing Renal Outcomes after Transcatheter versus Surgical Aortic Valve Replacement
Source: J Interv Cardiol. 2019 Apr 24;2019:3537256. doi: 10.1155/2019/3537256 (PMC6739771; doi:10.1155/2019/3537256)
Supplement: Supplementary Materials — eFigure 1: Funnel plot of the meta-analysis of the published studies reporting 30-day acute kidney injury in patients undergoing TAVR versus SAVR. TAVR: transcatheter aortic valve replacement; SAVR: surgical aortic valve replacement. eFigure 2: Funnel plot of the meta-analysis of the published studies reporting 30-day renal replacement therapy in patients undergoing TAVR versus SAVR. TAVR: transcatheter aortic valve replacement; SAVR: surgical aortic valve replacement. eFigure 3: Meta regression for age, gender, previous stroke, peripheral arterial disease, diabetes, chronic kidney disease, atrial fibrillation, and acute kidney injury and renal replacement therapy in patients undergoing TAVR versus SAVR. TAVR: transcatheter aortic valve replacement; SAVR: surgical aortic valve replacement. [file 3537256.f1.docx]

**Supplement 1. Review Protocol:**

**Meta-Analysis Comparing Renal Outcomes after Transcatheter versus Surgical Aortic Valve Replacement**

***Project timing***

**First draft of the protocol:** Oct 1st

**Final protocol:** Oct 20

**Search and data analysis completed:** 2 January 2018

**Final update of search and data analysis:** 2 January 2018

***Review question(s)***

To compare acute kidney injury outcomes of adults with severe aortic stenosis undergoing either transcatheter aortic valve implantation (TAVR) and surgical aortic valve replacement (SAVR)

***Searches***

We plan to search MEDLINE. Additional sources include Internet- based search engines and abstracts/presentations from major pertinent meetings. Prior systematic reviews and other studies references will be hand-searched to include relevant studies.

***Search Algorithm***

1. Surgical AND
2. Transcatheter OR percutaneous AND
3. Aortic AND
4. Valve replacement AND
5. Acute kidney injury OR acute renal failure

***Types of study to be included***

Studies meeting all the following inclusion criteria: 1. Randomized controlled trials or propensity-matched prospective observational studies comparing TAVR versus SAVR. 2. All adult patients > 18 years of age. 3. Study reporting acute kidney injury or acute renal failure outcomes. 4. All languages without restriction

***Condition or domain being studied***

Adult patients with severe aortic stenosis undergoing invasive management.

***Intervention(s), exposure(s):*** TAVR

***Comparator(s)/ control:*** SAVR

***Outcome(s)***

***Primary outcomes:***

- 30-day acute kidney injury or acute renal failure

***Secondary outcomes***:

- 30-day post-procedure renal replacement therapy

***Strategy for data synthesis***

The results of all studies will be pooled using the Mantel-Haenszel method and a DerSimonian-Laird random-effects model.

***Final update of search:*** 2 January 2018

1. Pubmed: 5,067

***Contact details for further information***

Mohamad Alkhouli, MD.

West Virginia University Heart & Vascular Institute

1 Medical Drive, Morgantown, WV 26505, USA.

Email: [Mohamad.Alkhouli@wvumedicine.org](mailto:Mohamad.Alkhouli@wvumedicine.org)

***Organizational affiliation of the review:*** None

***Review team***

*Kuldeep Shah*, MD, West Virginia University Heart & Vascular Institute.

*Tatiana Busu*, MD, West Virginia University Heart & Vascular Institute

***Funding sources/sponsors:*** None

***Conflicts of interest:*** None

***Language:*** English


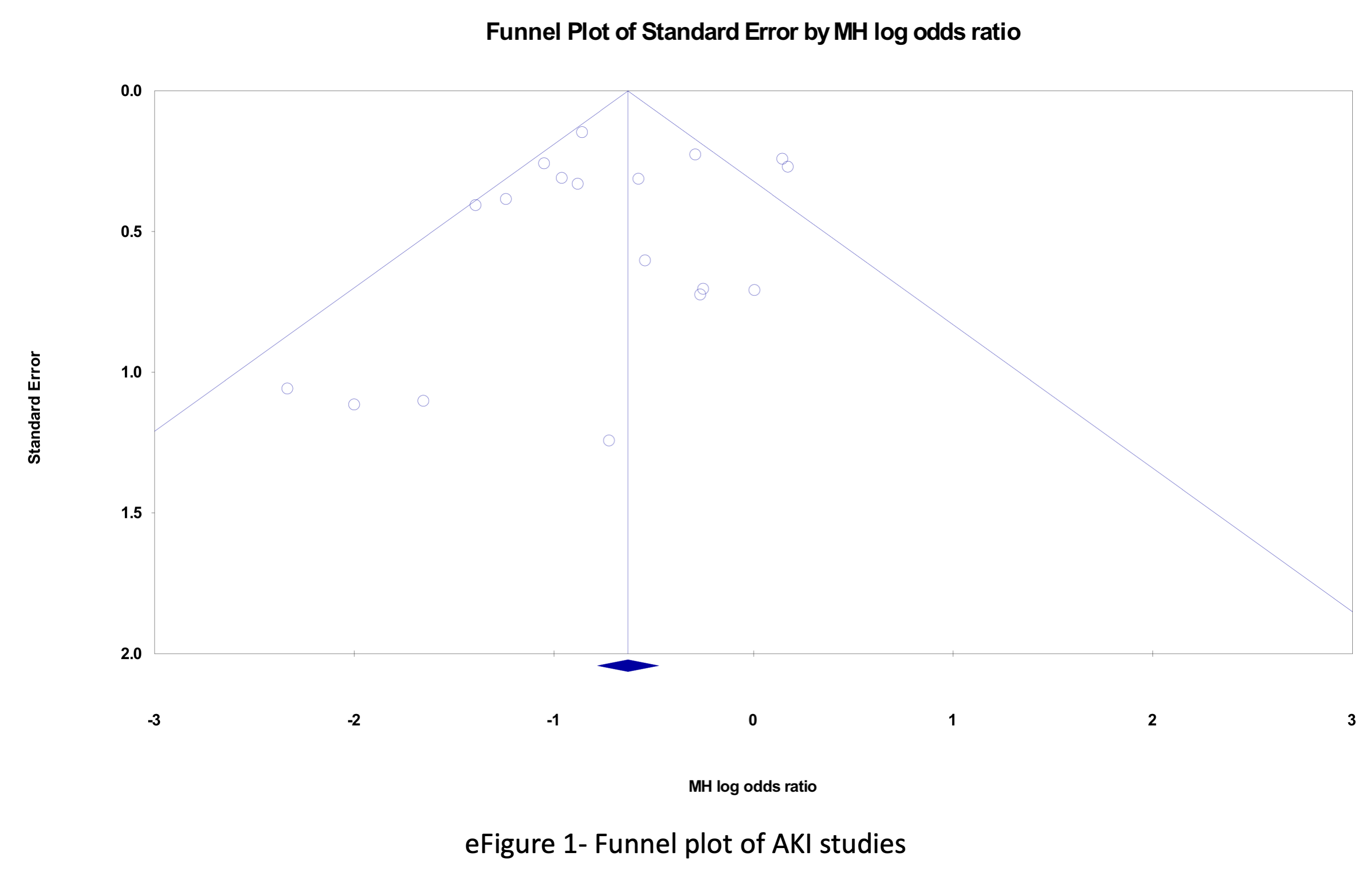


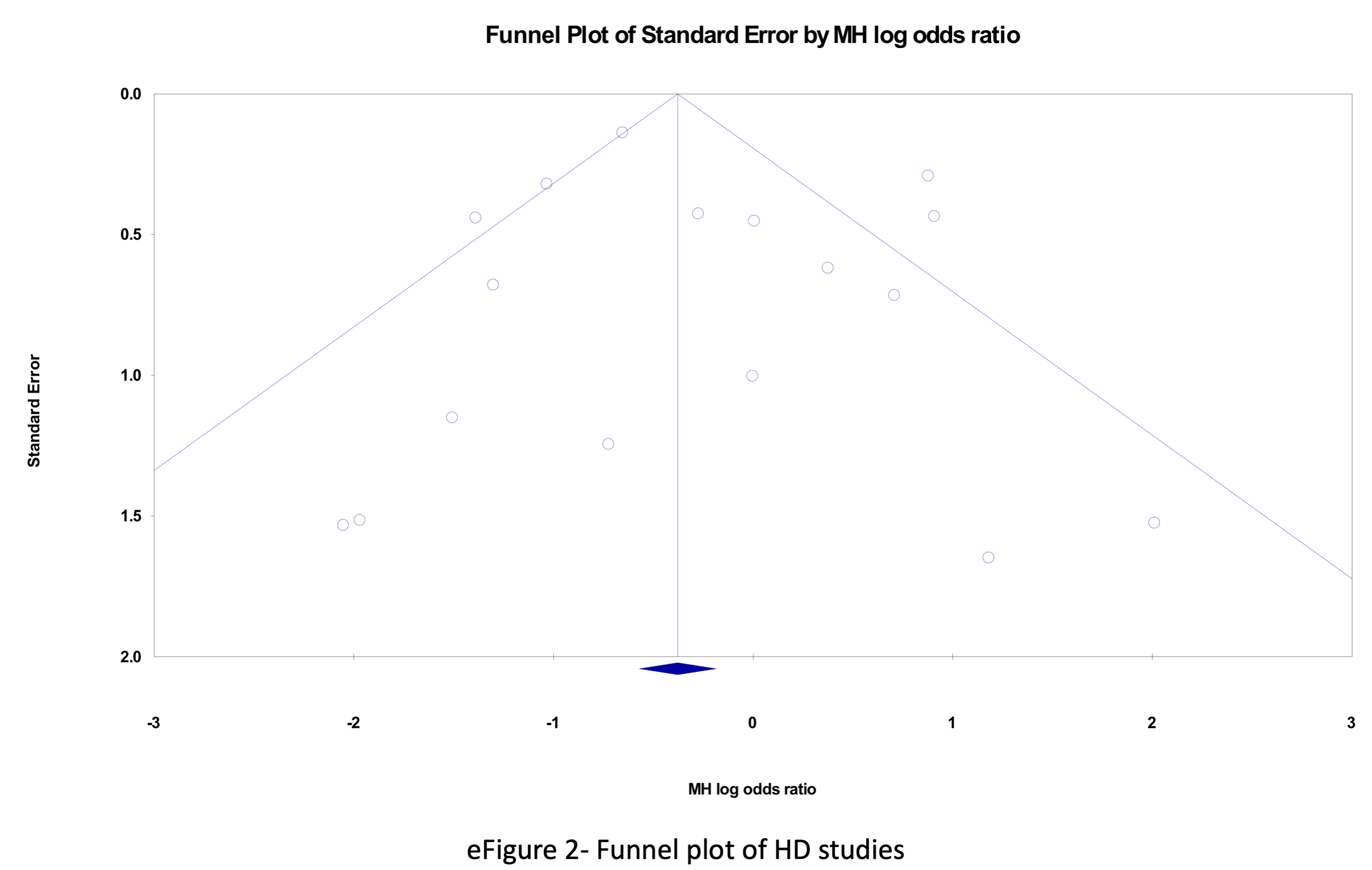


**eFigure-3; Meta-Regression of the Impact of Clinical Risk Factors on the Incidence of Acute Kidney Injury and Renal replacement therapy**

**1. Age*Acute Kidney Injury;**


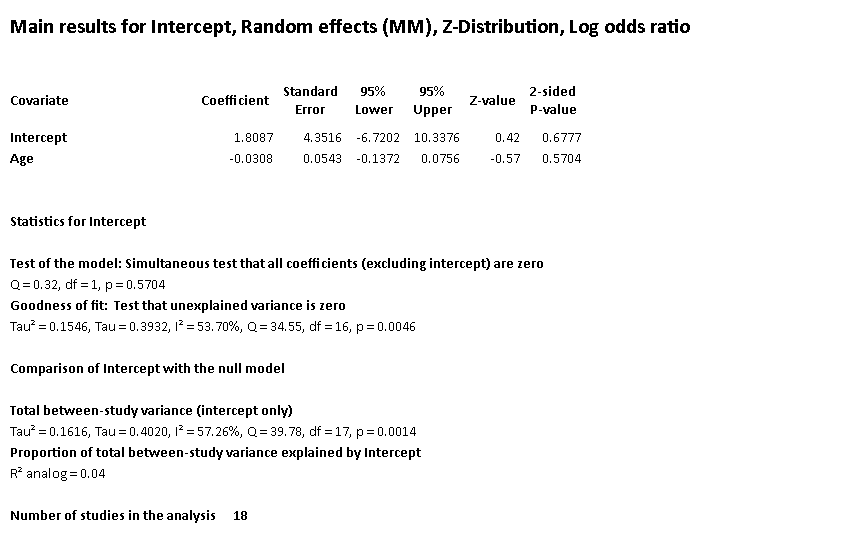


**2. Sex*Acute Kidney Injury;**


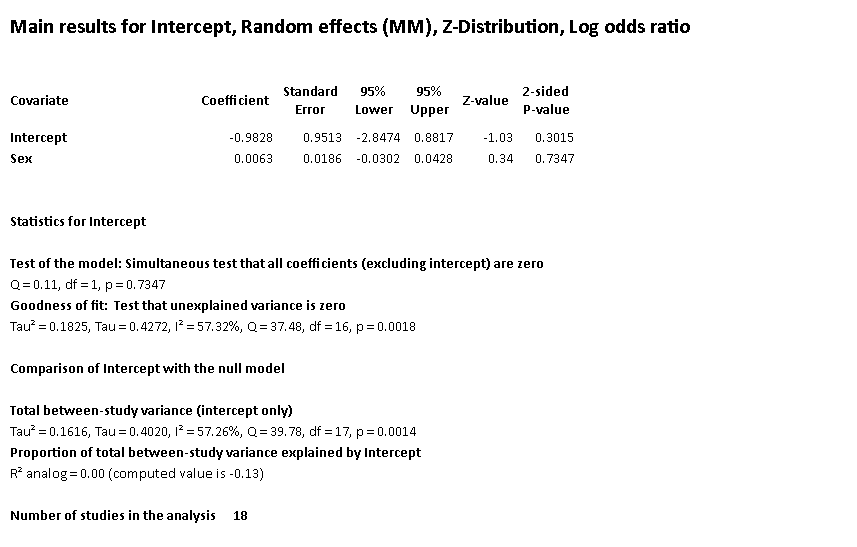


**3. Prior stroke * Acute Kidney Injury;**

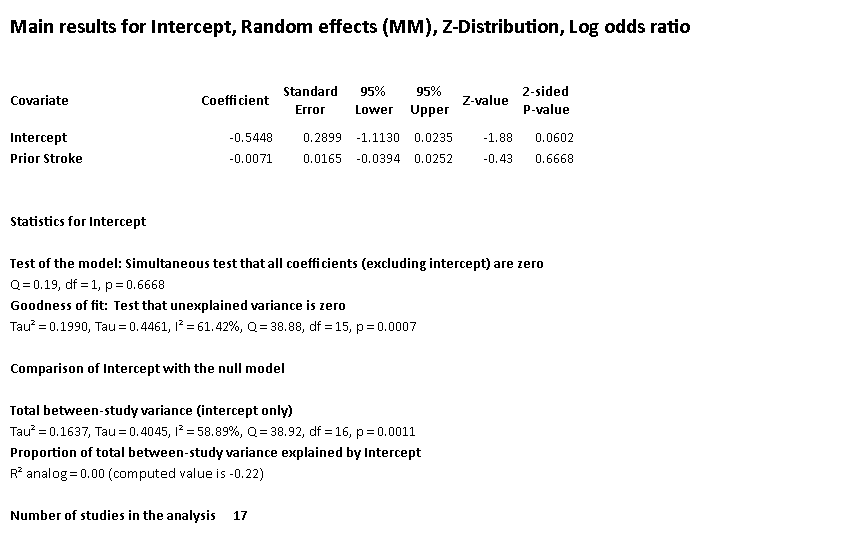


**4. Peripheral arterial disease * Acute Kidney Injury;**

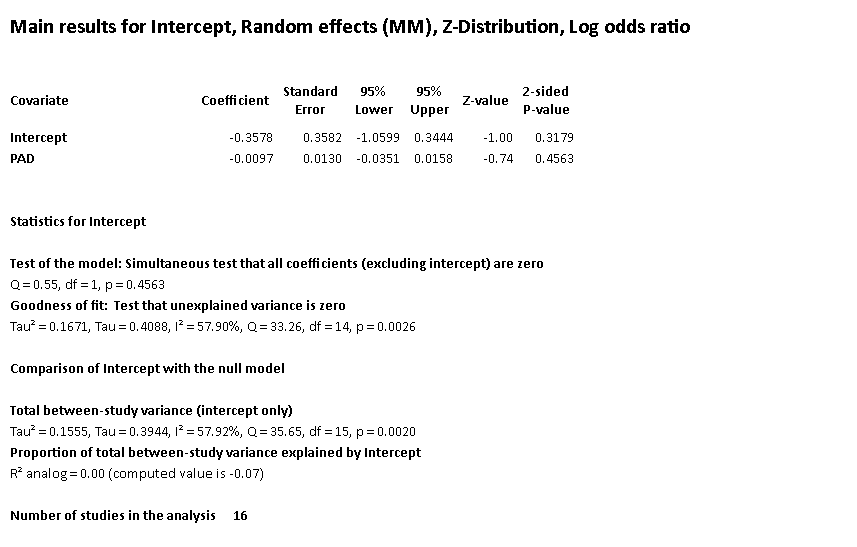


**5. Diabetes * Acute Kidney Injury;**

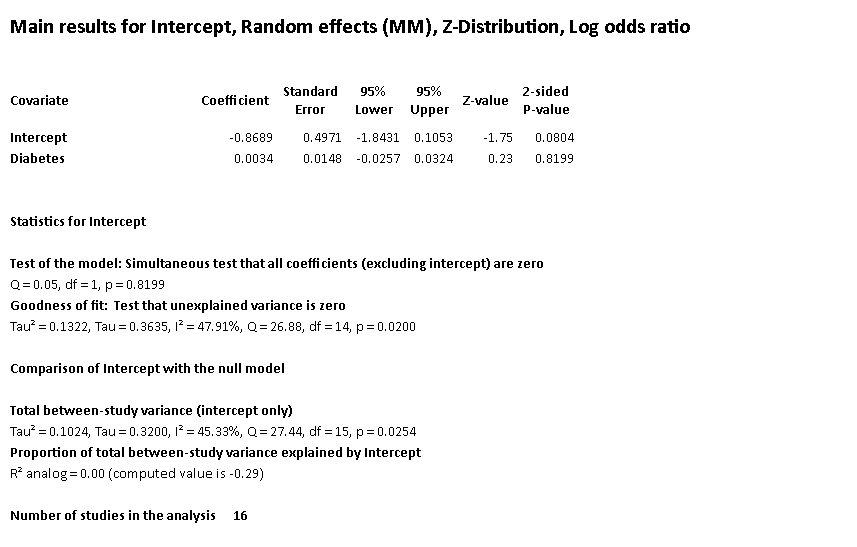


**6. CKD * Acute Kidney Injury;**

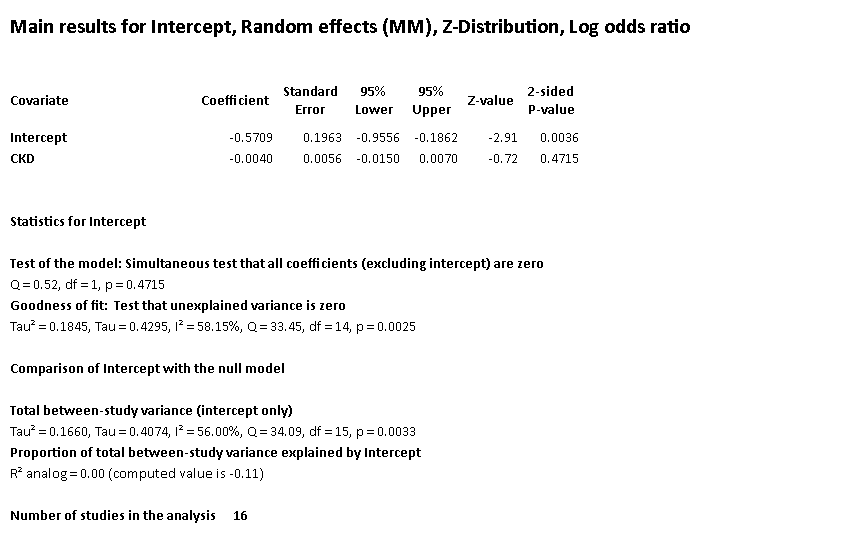


**7. Atrial Fibrillation * Acute Kidney Injury;**

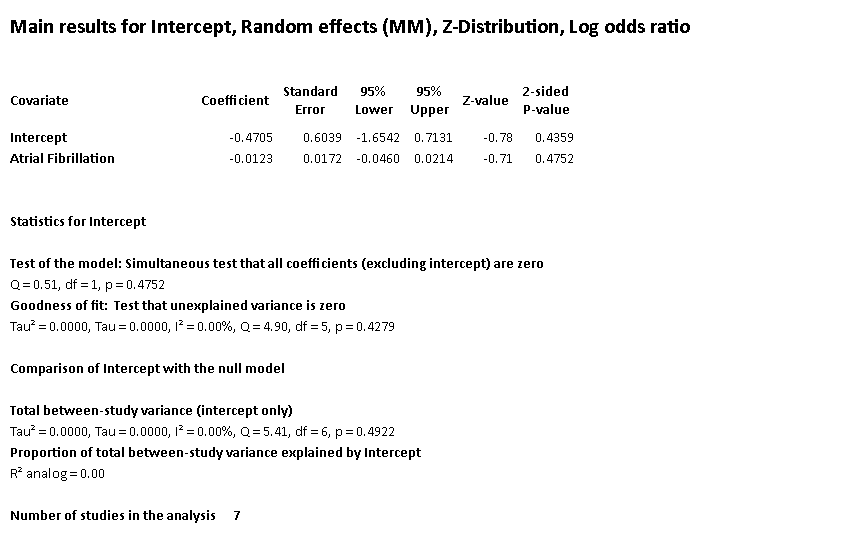


**8. Age * Renal Replacement therapy;**

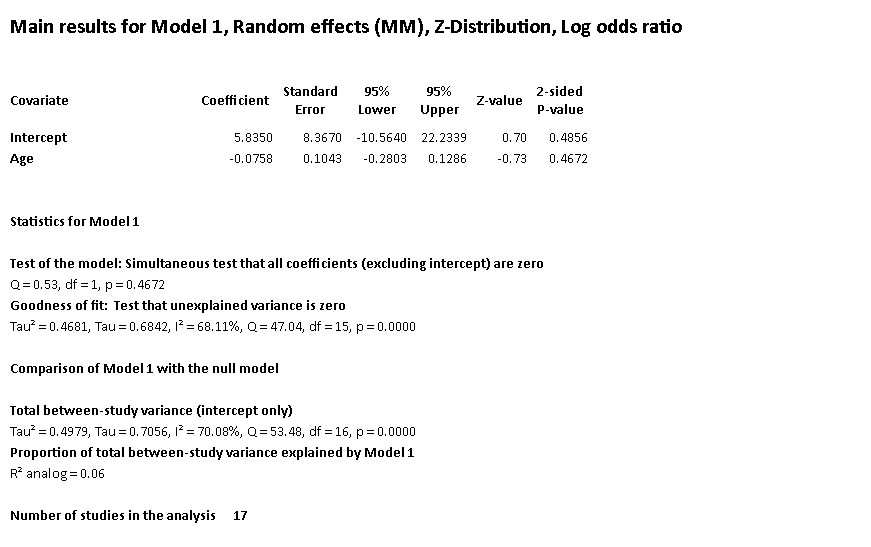


**9. Sex * Renal Replacement therapy;**

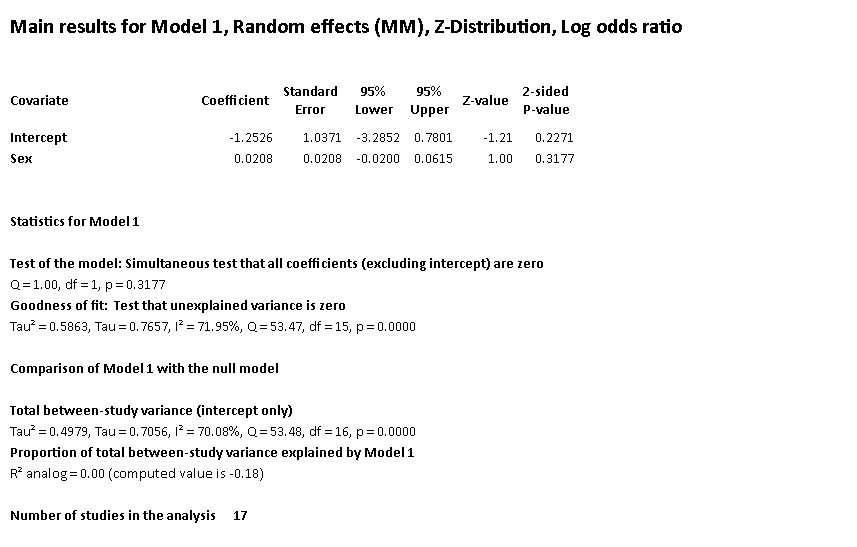


**10. Prior stroke * Renal Replacement therapy;**

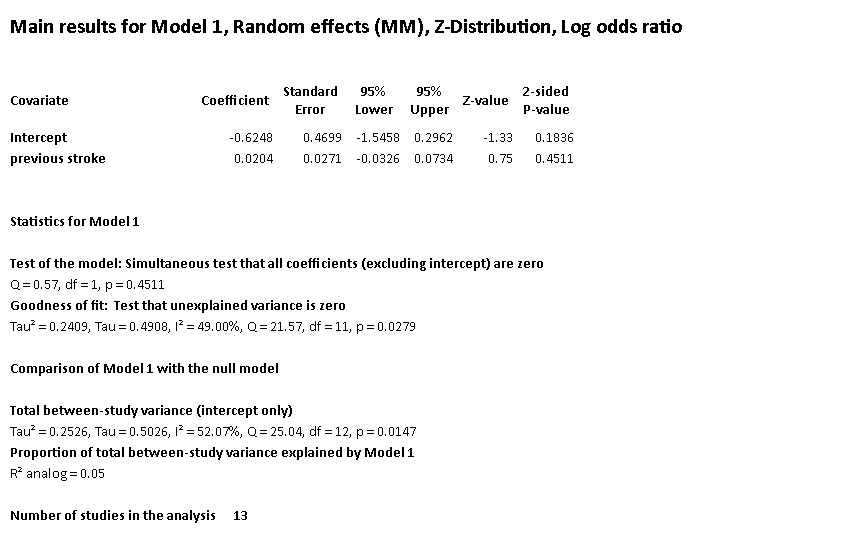


**11. Peripheral arterial disease * Renal Replacement therapy;**

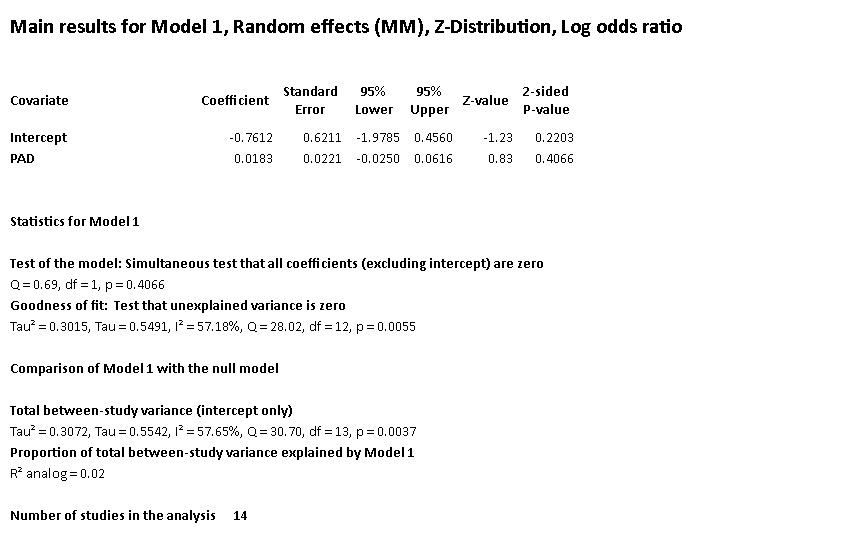


**12. Diabetes * Renal Replacement therapy;**

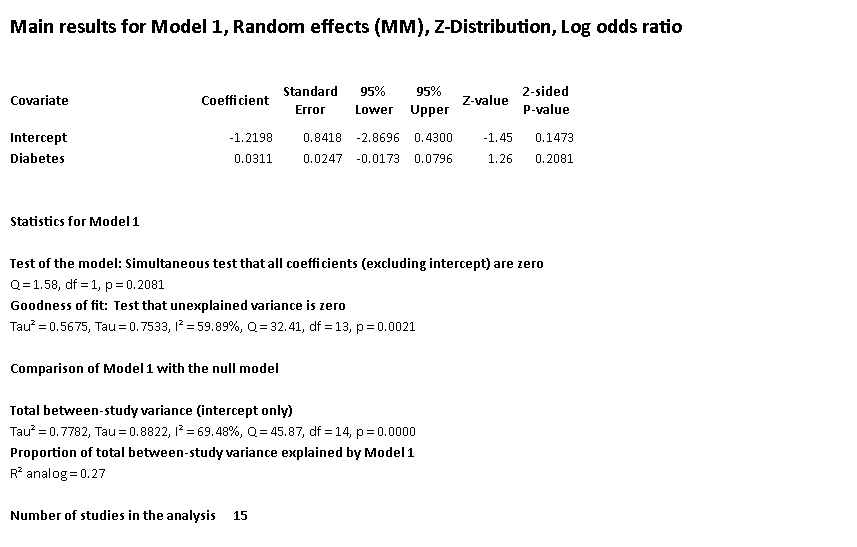


**13. CKD * Renal Replacement therapy;**

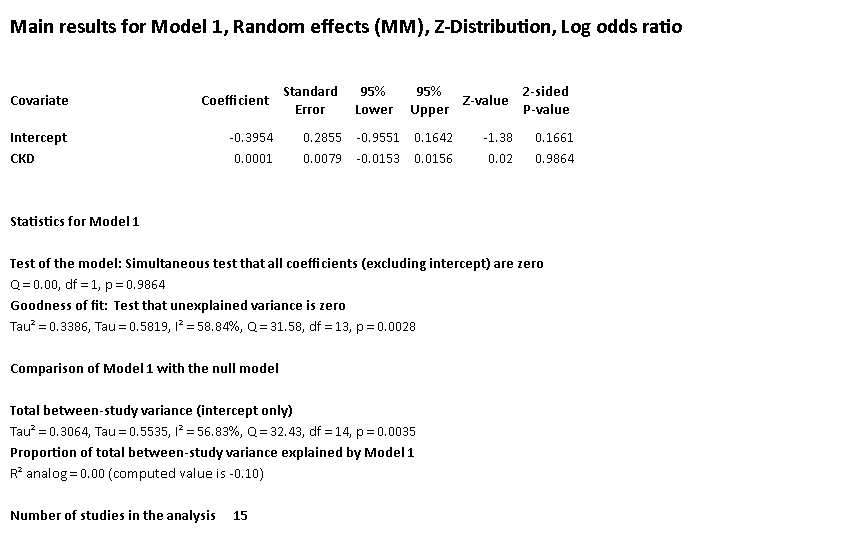


**14. Atrial Fibrillation * Renal Replacement therapy;**

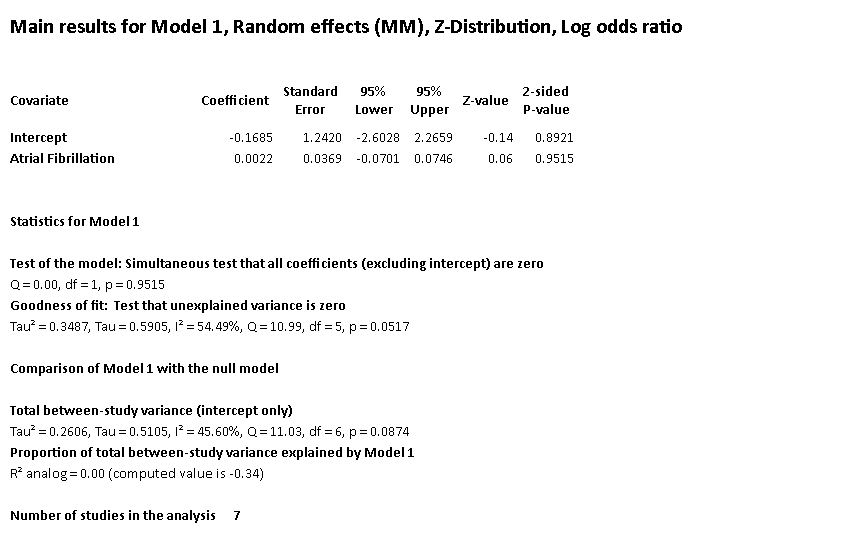


| Author/Year | Arm | N | Age | Male | CAD | CKD | Prior CABG | PAD | DM | COPD | LVEF | AF | Stroke/ TIA | NYHA III/IV | PHTN | Frail | STS Score | Euro-Score |
| --- | --- | --- | --- | --- | --- | --- | --- | --- | --- | --- | --- | --- | --- | --- | --- | --- | --- | --- |
| PARTNER 1  2011^1^ | SAVR | 351 | 84.5 | 56.7 | 76.9 | 7.0 | 44.2 | 41.6 | NR | 43.0 | 53.3 | 42.7 | NR | 94.0 | 36.4 | 17.6 | 11.7 | 29.2 |
|  | TAVR | 348 | 83.6 | 57.8 | 74.9 | 11.1 | 42.6 | 43.0 | NR | 43.3 | 52.5 | 40.8 | NR | 94.3 | 42.4 | 15.6 | 11.8 | 29.3 |
| STACCATO  2012^2^ | SAVR | 36 | 82.0 | 33.3 | NR | 0.0 | NR | 8.3 | 8.3 | 2.8 | 56.3 | NR | NR | NR | NR | NR | 3.4 | 10.3 |
|  | TAVR | 34 | 80.0 | 26.5 | NR | 2.9 | NR | 5.9 | 2.9 | 2.9 | 56.5 | NR | NR | NR | NR | NR | 3.1 | 9.4 |
| COREVALVE  2014^3^ | SAVR | 357 | 83.2 | 52.4 | 75.9 | 12.8 | 31.1 | 41.7 | 45.4 | 9.0 | 56.0 | 45.9 | 27.5 | 86.8 | NR | NR | 7.5 | 18.6 |
|  | TAVR | 390 | 83.1 | 53.1 | 75.4 | 12.2 | 29.5 | 41.1 | 34.9 | 13.3 | 56.9 | 40.9 | 25.4 | 85.6 | NR | NR | 7.3 | 17.7 |
| NOTION  2015^4^ | SAVR | 135 | 79.0 | 52.6 | NR | 0.7 | NR | 6.7 | 20.7 | 11.9 | NR | 25.6 | 16.3 | 45.5 | NR | NR | 3.1 | 8.9 |
|  | TAVR | 145 | 79.2 | 53.8 | NR | 1.4 | NR | 4.1 | 17.9 | 11.7 | NR | 27.8 | 16.6 | 48.3 | NR | NR | 2.9 | 8.4 |
| PARTNER 2A  2016^5^ | SAVR | 1021 | 81.7 | 54.4 | 66.5 | 5.2 | 25.6 | 32.9 | 34.2 | 30.0 | 76.1 | 35.2 | NR | 76.1 | NR | 61.1 | 5.8 | NR |
|  | TAVR | 1011 | 81.5 | 54.2 | 69.2 | 5.0 | 23.6 | 27.9 | 37.7 | 31.8 | 77.3 | 31.0 | NR | 77.3 | NR | 59.6 | 5.8 | NR |
| SURTAVI  2017^6^ | SAVR | 796 | 79.7 | 55.0 | 64.2 | 2.1 | 17.2 | 29.9 | 34.8 | NR | NR | 26.5 | 13.6 | 58.2 | NR | NR | 4.5 | 11.6 |
|  | TAVR | 864 | 79.9 | 57.6 | 62.6 | 1.6 | 16.0 | 30.8 | 34.1 | NR | NR | 28.1 | 13.3 | 60.2 | NR | NR | 4.4 | 11.9 |
| Bagur et al.  2010^7^ | SAVR | 104 | 74.0 | 48.0 | 18.0 | 100 | NR | NR | 40.0 | 22.0 | 55.0 | NR | NR | 15.0 | NR | NR | NR | 21.8 |
|  | TAVR | 119 | 83.0 | 41.1 | 70.0 | 100 | NR | NR | 27.0 | 25.0 | 56.0 | NR | NR | 55.0 | NR | NR | NR | 31.2 |
| Fusari et al.  2011^8^ | SAVR | 30 | 77.5 | 46.7 | 33.3 | 13.3 | NR | 30.0 | 6.7 | 33.3 | NR | 20.0 | 6.7 | NR | 10.7 | 16.7 | 6.1 | 11.7 |
|  | TAVR | 30 | 80.5 | 20.0 | 36.7 | 10.0 | NR | 40.0 | 23.3 | 30.0 | NR | 10.0 | 10.0 | NR | 10.7 | 23.3 | 6.6 | 14.7 |
| Stohr et al.  2011^9^ | SAVR | 175 | 79.3 | 44.1 | 58.0 | 2.3 | 0.0 | 24.4 | 33.1 | 33.7 | NR | NR | NR | NR | 12.2 | NR | NR | 16.7 |
|  | TAVR | 175 | 80.2 | 34.3 | 62.0 | 4.0 | 44.1 | 29.1 | 25.1 | 45.3 | NR | NR | NR | NR | 14.3 | NR | NR | 21.2 |
| Holzhey et al.  2012^10^ | SAVR | 167 | 80.5 | 35.3 | NR | NR | NR | NR | 44.3 | 13.8 | 56.3 | NR | NR | NR | 8.4 | NR | NR | 18.3 |
|  | TAVR | 167 | 79.8 | 35.3 | NR | NR | NR | NR | 39.5 | 13.8 | 56.0 | NR | NR | NR | 9.0 | NR | NR | 18.7 |
| Appel et al.  2012^11^ | SAVR | 45 | 77.0 | 49.0 | 22.0 | 69.0 | 0.0 | 9.0 | 18.0 | 7.0 | NR | 7.0 | NR | 82.2 | 9.0 | NR | 3.0 | 8.0 |
|  | TAVR | 45 | 81.0 | 49.0 | 16.0 | 56.0 | 20.0 | 18.0 | 18.0 | 7.0 | NR | 22.0 | NR | 84.4 | 20.0 | NR | 4.4 | 16.0 |
| Latib et al.  2012^12^ | SAVR | 111 | 79.4 | 44.1 | 45.9 | NR | NR | 34.2 | 21.6 | 22.5 | 53.6 | NR | NR | 69.4 | NR | NR | 4.6 | 24.4 |
|  | TAVR | 111 | 80.5 | 44.1 | 39.6 | NR | NR | 26.1 | 18.9 | 26.1 | 53.5 | NR | NR | 67.6 | NR | NR | 4.57 | 23.2 |
| D’onofrio et al. 2012^13^ | SAVR | 38 | 80.9 | 15.8 | 34.0 | 3.0 | NR | 13.2 | 21.0 | 13.2 | 60.0 | 15.8 | NR | 65.8 | NR | NR | NR | 13.7 |
|  | TAVR | 38 | 80.9 | 21.1 | 45.0 | 0.0 | NR | 18.4 | 26.0 | 21.1 | 60.0 | 13.1 | NR | 73.7 | NR | NR | NR | 14.8 |
| Wilbring et al. 2013^14^ | SAVR | 53 | 77.6 | 66.0 | 100 | 60.4 | 69.8 | 32.1 | 43.4 | 7.5 | NR | 35.8 | 15.1 | NR | 20.8 | NR | NR | 8.2 |
|  | TAVR | 53 | 78.1 | 65.0 | 100 | 66.0 | 73.6 | 47.2 | 52.8 | 9.4 | NR | 45.3 | 18.9 | NR | 18.8 | NR | NR | 8.8 |
| Papadopoulos et al. 2014^15^ | SAVR | 40 | 80.0 | 73.0 | 75.0 | 40.0 | NR | 27.0 | 35.0 | 20.0 | 47.0 | NR | NR | NR | 13.0 | NR | 9.0 | 17.0 |
|  | TAVR | 40 | 81.0 | 73.0 | 83.0 | 50.0 | NR | 33.0 | 42.0 | 23.0 | 48.0 | NR | NR | NR | 13.0 | NR | 11.0 | 25.0 |
| Santarpino et al.  2015^16^ | SAVR | 102 | 80.0 | 41.0 | NR | 33.0 | NR | 26.0 | 39.0 | 22.0 | 58.0 | NR | NR | NR | NR | NR | NR | 17.0 |
|  | TAVR | 102 | 79.0 | 43.0 | NR | 32.0 | NR | 16.0 | 36.0 | 23.0 | 54.0 | NR | NR | NR | NR | NR | NR | 18.0 |
| Schymik et al. 2015^17^ | SAVR | 216 | 78.2 | 51.4 | 48.1 | 3.2 | NR | 6.9 | NR | 8.8 | 62.0 | NR | NR | NR | 1.4 | NR | NR | 8.8 |
|  | TAVR | 216 | 78.3 | 46.3 | 48.1 | 3.2 | NR | 5.1 | NR | 9.3 | 62.2 | NR | NR | NR | 1.4 | NR | NR | 8.7 |
| Muneretto et al. 2015^18^ | SAVR | 204 | 80.0 | 52.0 | 24.0 | 18.1 | NR | 22.6 | 26.4 | 26.4 | 54.7 | NR | 10.7 | 61.2 | NR | NR | 8.3 | 19.2 |
|  | TAVR | 204 | 80.0 | 55.4 | 25.9 | 21.6 | NR | 21.0 | 30.3 | 27.4 | 54.6 | NR | 13.6 | 67.1 | NR | NR | 8.2 | 19.5 |
| Wendt et al.  2015^19^ | SAVR | 51 | 71.1 | 74.5 | NR | 21.0 | 64.7 | 29.4 | 43.1 | 33.3 | 29.9 | 25.5 | NR | NR | 13.7 | NR | 7.1 | 22.2 |
|  | TAVR | 62 | 78.7 | 69.4 | NR | 23.5 | 87.1 | 52.4 | 38.7 | 25.8 | 48.1 | 22.6 | NR | NR | 22.6 | NR | 12.1 | 36.4 |
| Thakkar et al.  2016^20^ | SAVR | 30 | 70.5 | 66.7 | NR | 63.3 | NR | 10.0 | 53.3 | 43.3 | NR | NR | NR | NR | NR | NR | NR | NR |
|  | TAVR | 30 | 71.7 | 73.3 | NR | 53.3 | NR | 20.0 | 53.3 | 23.3 | NR | NR | NR | NR | NR | NR | NR | NR |
| Thongprayoon et al.  2016^21^ | SAVR | 195 | 54.0 | 54.0 | NR | NR | 34.0 | NR | 42.0 | 50.0 | 57.0 | NR | 26.0 | 81.0 | NR | NR | 6.2 | NR |
|  | TAVR | 195 | 53.0 | 53.0 | NR | NR | 32.0 | NR | 37.0 | 53.0 | 57.0 | NR | 24.0 | 79.0 | NR | NR | 6.3 | NR |
| Biancari et al.  2016^22^ | SAVR | 144 | 79.4 | 38.8 | NR | 78.5 | NR | 8.3 | 4.2 | 26.4 | NR | NR | NR | 75.0 | 63.8 | NR | NR | 4.1 |
|  | TAVR | 144 | 79.0 | 37.5 | NR | 84.0 | NR | 9.0 | 3.5 | 24.3 | NR | NR | NR | 72.9 | 63.8 | NR | NR | 3.6 |
| Onorati et al. 2016^23^ | SAVR | 28 | 77.4 | 60.7 | NR | 39.3 | NR | NR | 42.9 | 25.0 | NR | NR | NR | 82.1 | 25.0 | NR | NR | 16.3 |
|  | TAVR | 28 | 78.8 | 75.0 | NR | 50.0 | NR | NR | 35.7 | 25.0 | NR | NR | NR | 78.6 | 25.0 | NR | NR | 14.3 |
| Fraccaro et al. 2016^24^ | SAVR | 415 | 83.7 | 40.0 | NR | 0.2 | NR | 17.3 | 19.3 | 17.6 | NR | NR | NR | 59.0 | 10.9 | 14.9 | NR | 9.9 |
|  | TAVR | 415 | 83.7 | 38.1 | NR | 0.7 | NR | 17.3 | 18.6 | 15.2 | NR | NR | NR | 58.5 | 13.2 | 15.2 | NR | 9.9 |
| Ailawadi et al. 2016^25^ | SAVR | 340 | 77.0 | 47.0 | NR | 6.4 | NR | 21.6 | 42.5 | NR | 55.0 | NR | NR | 62.0 | NR | NR | 6.2 | NR |
|  | TAVR | 340 | 81.0 | 49.4 | NR | 4.7 | NR | 9.0 | 39.0 | NR | 55.0 | NR | NR | 68.3 | NR | NR | 6.3 | NR |
| Brennan et al. 2017^26^ | SAVR | 4732 | 82.0 | 51.9 | NR | 3.9 | NR | 24.0 | NR | 22.5 | 55.0 | 34.2 | 11.1 | 70.5 | NR | NR | 5.8 | NR |
|  | TAVR | 4732 | 81.0 | 52.3 | NR | 3.8 | NR | 23.5 | NR | 22.8 | 55.0 | 33.2 | 10.7 | 71.8 | NR | NR | 5.5 | NR |

**eTable-1.**

**Detailed Baseline Characteristics of The Individual Included Studies**

N; number, CAD; coronary artery disease, CKD; chronic kidney disease, CABG; coronary artery bypass grafting, PAD; peripheral vascular disease, DM; diabetes mellitus, COPD; chronic obstructive pulmonary disease, LVEF; left ventricular ejection fraction, AF; atrial fibrillation, TIA; transient ischemic attack, NYHA; New-York heart association class, PHTN; pulmonary hypertension, STS; society of thoracic surgery; SAVR; surgical aortic valve replacement, TAVR; transcatheter aortic valve replacement, NR; not reported.
